# Intratumoral microorganisms in tumors of the digestive system

**DOI:** 10.1186/s12964-023-01425-5

**Published:** 2024-01-25

**Authors:** Mengjuan Xuan, Xinyu Gu, Yingru Liu, Li Yang, Yi Li, Di Huang, Juan Li, Chen Xue

**Affiliations:** 1 Department of Infectious Disease, The First Affiliated Hospital of Zhengzhou University, No. 1 Jianshe East Road, Erqi District, Zhengzhou, 450052 China; 2 Department of Oncology, The First Affiliated Hospital, College of Clinical Medicine, Henan University of Science and Technology, Luoyang, 471000 Henan China; 3 Department of Child Health Care, The Third Affiliated Hospital of Zhengzhou University, Zhengzhou, 450000 Henan China

**Keywords:** Intratumoral microbes, Digestive system tumors, Engineered bacterium, Treatment, Prognosis

## Abstract

Tumors of the digestive system pose a significant threat to human health and longevity. These tumors are associated with high morbidity and mortality rates, leading to a heavy economic burden on healthcare systems. Several intratumoral microorganisms are present in digestive system tumors, and their sources and abundance display significant heterogeneity depending on the specific tumor subtype. These microbes have a complex and precise function in the neoplasm. They can facilitate tumor growth through various mechanisms, such as inducing DNA damage, influencing the antitumor immune response, and promoting the degradation of chemotherapy drugs. Therefore, these microorganisms can be targeted to inhibit tumor progression for improving overall patient prognosis. This review focuses on the current research progress on microorganisms present in the digestive system tumors and how they influence the initiation, progression, and prognosis of tumors. Furthermore, the primary sources and constituents of tumor microbiome are delineated. Finally, we summarize the application potential of intratumoral microbes in the diagnosis, treatment, and prognosis prediction of digestive system tumors.

Video Abstract

**Supplementary Information:**

The online version contains supplementary material available at 10.1186/s12964-023-01425-5.

## Introduction

In 2019, the World Health Organization redefined the criteria for classifying digestive system tumors and included esophageal cancer (EC), gastric cancer (GC), colorectal cancer (CRC), pancreatic cancer (PC), and hepatocellular carcinoma (HCC) [1], among others in this classification. Tumor types are based on their molecular phenotype in addition to histopathological characteristics, reflecting the latest advancements in the understanding of digestive system tumors. Although considerable improvements have been achieved in traditional treatment modalities for neoplasms of the digestive system, patient prognosis remains poor. This happens because of inadequate early screening, limitations of current treatments, and the increased frequency of metastasis and recurrence of digestive system tumors [2, 3]. Consequently, identifying novel diagnostic biomarkers and treatment modalities, mitigating the emergence of antitumor drug resistance, and improving the outcomes and quality of life of patients is crucial for healthcare professionals.

The surface barriers of human body is populated by complex groups of bacteria, fungi, protozoa, and viruses [4]. The intestinal mucosa that lines the inner surface of various organs contains trillions of microbial species, and bacteria are the most dominant group among them [5, 6]. In 2015, Garrett et al. suggested that bacteria can promote tumorigenesis by disrupting cell growth homeostasis and regulating immune responses and drug metabolism [7]. The gastrointestinal tract acts as a repository of microorganisms in the human body and exerts a significant influence on the pathogenesis of various malignancies. Therefore, the gut microbiota can influence the development of tumors by acting as regulators of immune system stimulation [8]. In a word, gut microbes may trigger inflammation associated with cancer and impact the efficacy of various cancer therapies.

Several authors reported the presence of microbes in tumors in the 19th century [9]. However, the origin of the microbes was unclear because the lack of molecular detection techniques was not available to ascertain whether the detected microbes originated from within the tumor or from external contamination. Moreover, the low microbial density within tumors hindered research on their significance for over a century. The progress in the detection technology and an increased understanding of tumor microenvironments have validated the presence of intratumoral microorganisms in the past few years. The gastrointestinal system —the largest reservoir of microorganisms in the human body—is a comprehensive physiological network that interfaces with the external environment through the oral cavity and digestive tract. Notably, intratumoral microbes are most frequently detected in digestive system neoplasms [10–13].

In this review, we have detailed the fundamental attributes of microbiota present in tumors of the digestive system and the recent advancements in this field. Furthermore, we have innovatively elucidated the functions of typical microorganisms in gastrointestinal neoplasms and the underlying mechanisms by which they facilitate tumor initiation or inhibition of tumor progression. Finally, we focused on the prospective use of these microorganisms in the diagnosis, therapy, and predicting the outcomes of gastrointestinal malignancies. We express our genuine anticipation that our analysis will provide the baseline to explore novel diagnostic and therapeutic approaches for malignancies of the gastrointestinal tract.

## Detection methods for intratumoral microbes and their general characteristics

**Fig. 1:**
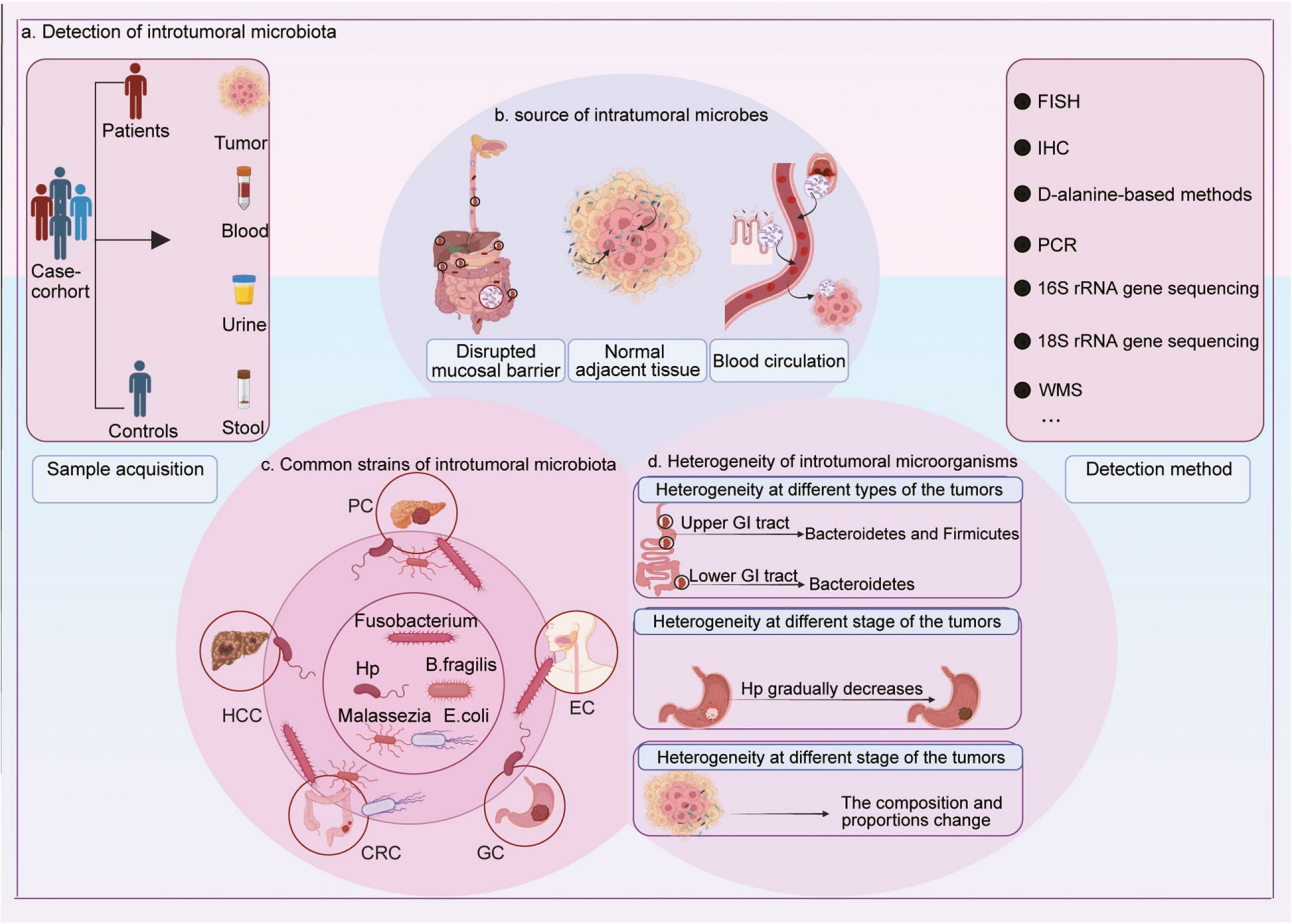
Intratumoral microbes: Detection methods and general characteristics. **a**. Several methods are used to detect microorganisms in tumors, and next-generation sequencing technology is the primary detection method.**b**. Microbes can enter and colonize tumor tissue by crossing damaged mucosal barriers, migrating from adjacent normal tissues, or through the circulatory system.**c**. *Fusobacterium*, *Bacteroides fragilis*, *Escherichia coli*, *Helicobacter pylori*, and *Malassezia* are the common microbes in digestive system tumors.**d**. Microbes show substantial heterogeneity in various types of tumors and during different stages of the same tumor. Moreover, microbial populations in tumors differ from those in neighboring healthy tissues

The continual advancements in technology have enabled us to trace the origin of tumor-associated bacteria, determine the specific composition of intratumoral microbiota, and gauge the abundance of microorganisms in different types of digestive system tumors (Fig. 1).

### Detection methods for microbes in digestive system tumors

Next-generation sequencing has emerged as a promising technique to obtain a comprehensive microbial profile of the sample without the requirement for culturing [14]. Deep sequencing techniques involving universal marker gene amplicons are frequently utilized for investigating microbiomes, and 16 S rRNA sequencing and shotgun metagenomic sequencing are the most commonly used methods [15, 16]. 16 S rRNA sequencing is an efficient technique for characterizing bacterial diversity but with less detailed functional information. Whole metagenomic shotgun sequencing has several advantages over 16 S rRNA sequencing, including identification of both non-bacterial and bacterial taxa, strain/species level resolution, and functional annotation of the microbiota [17, 18]. In addition to sequencing techniques, intratumoral microorganisms can also be detected using fluorescent labeling of microbial antigens, genetic material, or metabolites [14, 19]. Overall, the use of advanced detection methods has enabled the rapid identification of microorganisms present within tumor tissues.

### Sources of microbiota in digestive system tumors

Bacteria can infiltrate gastrointestinal neoplasms by passing through disrupted mucosal barriers, migrating from adjacent normal tissue, or blood circulation [20]. Tjalsma et al. [21]. (2012) proposed a unique bacterial driver–passenger model of CRC pathogenesis. They suggested that specific intratumoral bacteria (drivers) can facilitate the infiltration of other gut microorganisms (passengers) into the tumor microenvironment. Interestingly, fluorescently labeled *Enterococcus faecalis* and GFP-labeled *Escherichia coli* (*E. coli*) were detected within the pancreatic microenvironment in mice [22]. The use of advanced detection methods has highlighted the existence of bacteria in otherwise normally sterile organs, including the pancreas [23]. Notably, the composition of bacterial detected in PC tissues is extremely similar to that observed in healthy adjacent pancreatic tissues [24, 25]. Abed et al. reported that *Fusobacterium nucleatum (F. nucleatum)*, a major constituent of the human oral microbiome, uses a hematogenous pathway to infiltrate colon adenocarcinomas [26].

### Microbial diversity in digestive system cancers

The utilization of next-generation sequencing technology enables the identification of all microorganisms, without the requirement of culturing, while enabling the identification of bacterial species based on their genetic profiles [16]. Bacterial colonization within tumors of the digestive system is a well-documented phenomenon, and these bacteria originate from diverse sources and show significant species-level variability [11, 27, 28]. Several common bacteria have been identified in digestive system tumors. *Helicobacter pylori* (*H. pylori*), a recognized risk factor for chronic gastritis and stomach cancer [29], is present not only in the cases of gastric malignancies but also in those of HCC and PC [23, 30]. The bacterial genus *Fusobacterium*, frequently detected in CRC [31], has also been associated with an unfavorable prognosis in PC [32]. *Bacteroides fragilis (B. fragilis)* colonizes the tumor microenvironment of colitis-associated CRC and can cause DNA damage [33]. The presence of *E. coli* in CRC can disrupt the gut vascular barrier and promote the development of a conducive microenvironment for liver metastases [34]. The diversity of bacteria present in digestive system tumors is extensive, thereby necessitating comprehensive investigations involving large sample sizes.

### Microbial heterogeneity within digestive system tumors

The composition of bacterial species in each tumor is considerably variable. The microbiome in the cohorts of patients with upper gastrointestinal tract EC and GC predominantly comprised Bacteroidetes and Firmicutes at the phylum level. In contrast, only Bacteroidetes were dominant in the samples collected from the patients with lower gastrointestinal tract cancers [27]. Additionally, bacterial diversity was significantly different between cancerous and adjacent non-cancerous tissues [27, 35]. Yuan and colleagues reported that *Porphyromonas gingivalis* was predominant and frequently present in the samples of EC and esophageal dysplasia tissues [36]. However, the bacterium was infrequently present in matched noncancerous segments. Similarly, *Fusobacterium* species were highly abundant in human colonic adenomas compared to the adjacent normal tissues [37].

Further, the composition of bacteria can vary during different phases of tumor progression. As the GC advances, *H. pylori* gradually diminishes in the tumor tissues and eventually disappears [38, 39]. *B. fragilis* toxin (BFT) facilitates inflammation, and advanced-stage CRC tissues show high BFT positivity compared to early-stage counterparts [33]. Nevertheless, it is unclear whether this happens due to an increased number of bacterial colonies in late-stage CRC or an increased secretion of toxins. Overall, the variations in microbial diversity among distinct tumors, cancerous versus non-cancerous tissues, and different stages of tumor progression can be exploited to develop new diagnoses and treatment strategies for digestive system tumors.

Furthermore, the relative abundance and source of bacteria within digestive tumors also exhibits substantial heterogeneity. In the microenvironment of HCC, there was a significant alteration in the abundance of microorganisms, including a reduction in the prevalence of *Pseudomonadaceae* and an increase in the prevalence of *Agrobacterium and Rhizobiaceae* [40]. Specially, a positive and linear correlation was observed between the presence of *Pseudomonadaceae* and the prognosis of patients with HCC. *Bifidobacteria*, as intestinal commensal bacteria, can infiltrate CRC tissues through disrupted intestinal intestinal barrier, leading to elevated abundance of *Bifidobacteria* [21, 41, 42]. Additionally, the secretion of lactic acid and acetic acid by *Bifidobacteria* enhances the growth and immune evasion of CRC, thereby negatively impacting prognosis [43]. Further investigations are required to identify variations in the abundance and source of microorganisms and their influence on the prognosis of distinct subtypes of digestive tumors.

## Intratumoral microbiome in different digestive system tumors

**Fig. 2:**
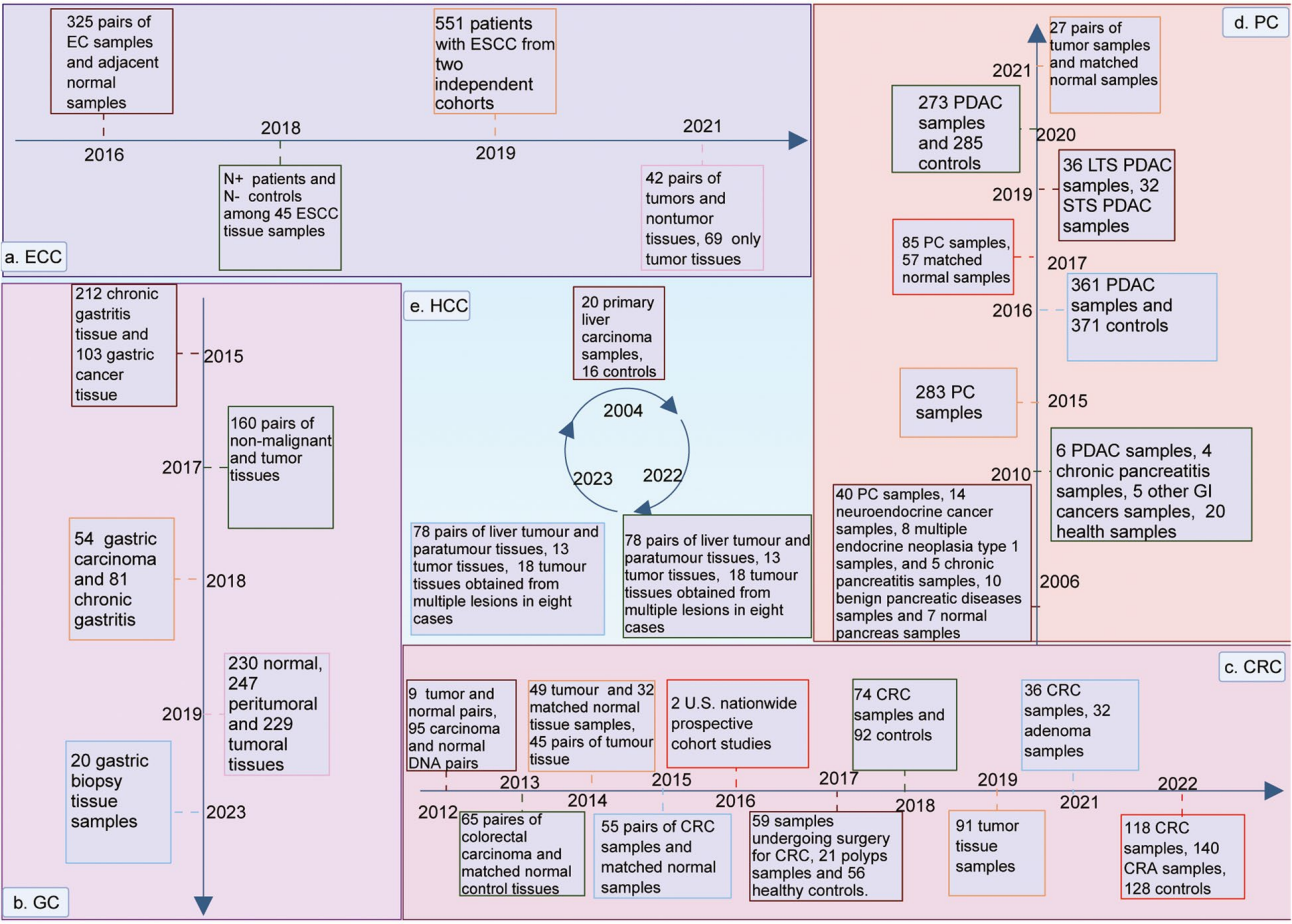
Research studies on intratumoral microbiomes in different digestive system tumors. The number of samples included in different studies on bacteria in tumors of the digestive system is listed in a chronological order using a time axis

The role of microorganisms in the development, detection,  prognostication, and therapy of cancer has been a matter of debate [44–46]. Next-generation sequencing technology has provided an unparalleled opportunity to investigate the genomes of tumor cells and hosts, as well as the diverse microorganisms that inhabit living organisms [47]. The following sections summarize the published studies on microorganisms identified in tumors of the digestive system using novel sequencing techniques (Table 1; Fig. 2).

**Table 1:** Intratumoral microbiome in different digestive system tumors

Cancer type	Year	Included samples	Methods	Enriched microorganism	Reduced microorganism	Implications	Ref
Esophageal cancer	2016	325 pairs of EC samples and adjacent normal tissue samples	qPCR	*Fusobacterium nucleatum*	/	predicting shorter survival	[48]
	2018	45 esophageal squamous cell carcinoma tissue samples from N+ (positive lymph node) patients and N- (negative lymph node) controls	16 S rRNA sequencing	*Phylum Bacteroidetes*, *Firmicutes*, and *Spirochetes*, *Streptococcus*, *Prevotella*	*Proteobacteria*	associated with unfavorable survival	[49]
	2019	551 patients with esophageal squamous cell carcinoma from two independent cohorts	qPCR	*Fusobacterium nucleatum*	/	predicting poor recurrence-free survival	[50]
	2019	16 controls, 14 Barrett’s esophagus without dysplasia, 6 low-grade dysplasia, 5 high-grade dysplasia, and 4 esophageal adenocarcinoma samples	16 S rRNA sequencing	*Proteobacteria, Enterobacteriaceae, Akkermansia, muciniphila*	*Firmicutes*, *Veillonella*	promoting carcinogenesis	[12]
	2021	120 esophageal squamous cell carcinoma resected samples and 30 pre-treatment biopsy samples	qPCR	*Fusobacterium nucleatum*	/	inducing chemoresistance	[51]
	2021	42 pairs of tumors and nontumor tissues, 69 only tumor tissues	16 S rRNA sequencing	*Bacteroidetes*, *Fusobacteria*, *Spirochetes*	*Butyrivibrio*, *Lactobacillus*	predicting prognosis and metastasis	[52]
	2021	40 tumor samples of esophageal squamous cell carcinoma, 20 tumor samples of esophageal adenocarcinoma, and 22 adjacent normal samples	/	*Firmicutes*, *Fusobacteria*	*Proteobacteria*	associated with clinical features	[28]
Gastric cancer	2015	212 chronic gastritis tissue and 103 gastric cancer tissue	qPCR	/	/	increasing bacterial load	[53]
	2017	160 pairs of non-malignant and tumor tissues	16 S rRNA sequencing	*Bacteriodetes*, *Firmicutes*, *Fusobacteria*, *Spirochetes*	*Proteobacteria*	altering non- *Helicobacter pylori* bacteria abundance	[39]
	2018	54 gastric carcinoma and 81 chronic gastritis tissues	16 S rRNA sequencing, qPCR	*Actinobacteria*, *Firmicutes*	*Bacteroidetes*, *Fusobacteria*	distinguishing between gastritis and gastric cancer	[54]
	2019	230 normal, 247 peritumor, and 229 tumor tissues	16 S rRNA sequencing	*Prevotella melaninogenica*, *Streptococcus anginosus*, *Propionibacterium acnes*	*Helicobacter pylori*, *Prevotella copri*, *Bacteroides uniformis*	/	[55]
	2023	20 gastric biopsy tissue samples	Whole metagenomic shotgun sequencing	/	/	revealing changes in composition and function	[56]
Colorectal cancer	2012	11 pairs of colorectal carcinoma and adjacent normal tissue specimens	qPCR	*Fusobacterium*	/	revealing the prevalence of *Fusobacterium* infection in colorectal cancer	[57]
	2012	9 tumor and normal pairs, 95 carcinoma and normal DNA pairs	whole genome sequences, qPCR, 16 S rDNA sequencing, FISH	*Fusobacterium*	*Bacteroidetes, Firmicutes*	revealing *Fusobacterium* associated with colorectal cancer	[58]
	2013	65 pairs of colorectal carcinoma and matched normal control tissues	Metatranscriptomic analysis	*Fusobacterium*, *Leptotrichia*, *Campylobacter spp*	/	revealing *Fusobacterium* associated with colorectal cancer	[59]
	2014	49 tumor and 32 matched normal tissue samples, 45 pairs of tumors tissue and matched normal tissue samples, 28 tumor and 52 adenoma samples	qPCR	*Fusobacterium*	/	promoting tumor progression	[60]
	2015	31 cancerous tissues and 20 adjacent non-cancerous tissues, 15 proximal colon cancer tissues and 16 distal colon cancer tissues, 15 healthy proximal colon tissues and 15 healthy distal colon tissues	16 S rRNA sequencing	*Firmicutes*, *Fusobacteria*, *Lactococcus*, *Fusobacterium*	*Proteobacteria*, *Pseudomonas*, *Escherichia*– *Shigella*	associated with colorectal cancer risk	[61]
	2015	55 pairs of colorectal cancer samples and matched normal samples	qPCR	enterotoxigenic *Bacteroides fragilis*, pks-positive *E. coli*, *Fusobacterium* spp.	/	associated with clinicopathological features	[62]
	2015	44 pairs of colorectal cancer samples and matched normal samples	16 S rRNA gene sequencing, qPCR	*Proteobacteria*, *Fusobacteria*	*Firmicutes*, *Bacteroidetes*	promoting tumor progression	[63]
	2016	2 U.S. nationwide prospective cohort studies	qPCR	*Fusobacteria*	/	predicting shorter survival	[64]
	2017	59 samples from patients undergoing surgery for colorectal cancer, 21 polyp samples, and 56 healthy controls	16 S rRNA sequencing, qPCR	*Bacteroidetes Cluster 2*, *Firmicutes Cluster 2*, *Pathogen Cluster*, Prevotella Cluster	*Bacteroidetes Cluster 1*, *Firmicutes Cluster 1*	associated with distinct mucosal gene-expression profiles	[65]
	2017	160 microsatellite instability-high colorectal cancer samples	qPCR	*Fusobacterium nucleatum*	/	modulating the immune microenvironment	[66]
	2017	11 pairs of fresh-frozen tumor and liver metastases samples, 77 fresh-frozen tumor samples, published data from 430 resected fresh-frozen colorectal cancer and 201 resected fresh-frozen hepatocellular carcinoma samples, 101 pairs of formalin-fixed paraffin-embedded colorectal cancer and liver metastases, 13 colorectal cancer samples for xenograft	qPCR, 16 S rRNA sequencing	*Fusobacterium*	/	associated with liver metastasis	[67]
	2018	5 FAP resected tissues and 1 juvenile polyposis syndrome resected tissue, 20 controls	16 S rRNA sequencing, FISH	*Bacteroides fragilis, Escherichia coli*	*Fusobacteria*	inducing tumorigenesis	[68]
	2018	74 colorectal cancer samples and 92 controls	shotgun metagenomic analyses	*Bacteriophage*	/	predicting colorectal cancer progression	[69]
	2018	1014 tumor tissue samples	qPCR	*Fusobacterium nucleatum*	/	promoting tumor progression	[70]
	2019	184 colorectal cancer samples, 197 adenoma samples, 204 controls	shotgun metagenomic sequencing	*Malasseziomycetes*	*Saccharomycetes*, *Pneumocystidomycetes*	revealing colorectal cancer-associated mycobiome dysbiosis	[71]
	2019	91 tumor tissue samples	qPCR	*Fusobacterium nucleatum*	/	predicting poor prognosis	[72]
	2021	36 colorectal cancer samples, 32 adenoma samples	16 S rRNA sequencing	/	/	revealing microbial community heterogeneity	[73]
	2021	excised colons from mice, human colonic adenomas	qPCR	/	*Fusobacterium nucleatum*	inhibited by aspirin	[74]
	2022	1,368 samples from 7 publicly available cohorts and one new Chinese cohort	shotgun metagenomic sequencing	*Candida pseudohaemulonis*, *Aspergillus ochraceoroseus*, *A. rambellii*, *Malassezia globosa*	*Aspergillus niger*, *Macrophomina phaseolina*, *Talaromyces islandicus*, *Sistotremastrum niveocremeum*	revealing changes in fungal microbiome colorectal cancer	[75]
	2022	118 colorectal cancer samples, 140 colorectal adenomas samples, 128 controls	GC-TOFMS, whole-genome shotgun sequencing	/	/	using intestinal metabolites to diagnose colorectal cancer	[76]
	2022	116 samples of adenocarcinomas, paired adenomatous polyps, and paired normal colon tissues	16 S rRNA sequencing, qPCR	*B. fragilis*, *Fusobacterium*, *Prevotella intermedia*	/	predicting malignant transformation, migration and invasion	[77]
Pancreatic cancer	2006	40 pancreatic cancer samples, 14 neuroendocrine cancer samples, 8 multiple endocrine neoplasia type 1 samples, and 5 chronic pancreatitis samples; 10 benign pancreatic disease samples and 7 normal pancreatic samples	PCR	*Helicobacter pylori*	/	revealing the association between *Helicobacter pylori* and PC	[23]
	2010	6 pancreatic ductal adenocarcinoma samples, 4 chronic pancreatitis samples, 5 other gastrointestinal cancers samples, 20 healthy samples	PCR	/	/	revealing the impossibility of direct infection	[78]
	2015	283 pancreatic cancer samples	qPCR	*Fusobacterium*	/	predicting poor prognosis	[32]
	2016	361 pancreatic ductal adenocarcinoma samples and 371 controls	16 S rRNA sequencing	*P. gingivalis*, *A. actinomycetemcomitans*, *Fusobacteria*	/	promoting carcinogenesis of pancreatic cancer	[79]
	2017	113 pancreatic ductal adenocarcinoma samples, 20 normal pancreatic tissue samples	qPCR, 16 S rRNA sequencing, immunohistochemistry	*Gammaproteobacteria*	/	mediating gemcitabine resistance	[80]
	2018	7 normal samples, 4 pancreatitis samples, 16 pancreatic ductal adenocarcinoma samples	16 S rRNA sequencing	/	/	revealing the presence of microbes in pancreas	[24]
	2019	41 mild acute pancreatitis samples, 59 moderately severe acute pancreatitis samples, 30 severe acute pancreatitis samples, 35 healthy controls	16 S rRNA sequencing	*Acinetobacter*, *Stenotrophomonas*, *Geobacillus*	*Bacteroides*, *Alloprevotella*, *Blautia*, *Gemella*	revealing the presence of microbes in pancreas	[81]
	2019	83 samples of pancreatic cancer patients receiving neoadjuvant therapy, 89 samples of pancreatic cancer patients who underwent surgery	/	Enterococci, *Klebsiella*	/	revealing the alteration in the biliary microbiome	[82]
	2019	36 long-term survival pancreatic adenocarcinoma samples, 32 short-term survival long-term survival samples	16 S rRNA sequencing	*Alphaprotebacteria Sphingobacteria*, *Flavobacteria*	*Clostridia*, *Bacteroides*	influencing pancreatic cancer outcomes	[11]
	2019	/	18 S rRNA sequencing, FISH	*Malassezia*	/	promoting pancreatic cancer progression	[83]
	2020	273 pancreatic adenocarcinoma samples and 285 controls	16 S rRNA sequencing	*Enterobacteriaceae*, *Lachnospiraceae G7*, *Bacteroidaceae*, *Staphylococcaceae*	*Haemophilus*	revealing the association between oral microbiota and pancreatic cancer	[84]
	2020	15 pairs of pancreatic adenocarcinoma samples and matched normal samples	16 S rRNA sequencing	*Tepidimonas*, *Akkermansia*	*Leuconostoc*, *Sutterella*	revealing the diversity of the microbiome in pancreatic adenocarcinoma	[85]
	2020	solid (duodenal mucosa, pancreatic tumor tissue, and stool) and liquid (bile and drainage fluid) biopsy samples from 10 patients	16 S rRNA sequencing	*Enterococcus*	/	associated with postoperative pancreatic fistula	[86]
	2020	74 pancreatic adenocarcinoma samples, 98 pancreatic cyst samples, 134 normal pancreas controls	16 S rRNA sequencing, 18 S rRNA sequencing	*Bifidobacterium*, *Fusobacteria*, *Rothia*	/	altering duodenal fluid microbiome profile in pancreatic adenocarcinoma	[87]
	2021	27 pairs of tumor samples and matched normal samples	16 S rRNA sequencing, qPCR	*Enterobacteriaceae*	/	promoting bacterial colonization of pancreatic adenocarcinoma tissues	[25]
Hepatocellular carcinoma	2004	20 primary liver carcinoma samples, 16 controls	16 S rRNA sequencing, PCR	/	/	detecting the 16 S rDNA of *Helicobacter pylori* in liver cancer tissue	[30]
	2022	156 samples: 28 normal liver, 64 peritumor, and 64 hepatocellular carcinoma tissues	16 S rRNA sequencing	*Patescibacteria*, *Proteobacteria*, *Bacteroidota*, *Firmicutes*, *Actinobacteriota*	/	revealing the intratumoral bacterial metataxonomic signature in hepatocellular carcinoma	[88]
	2023	78 pairs of liver tumor and paratumor tissues, 13 tumor tissues, 18 tumor tissues obtained from multiple lesions in 8 cases	FISH, 16 S rDNA sequencing	*Proteobacteri*a, *Actinobacteria*	*Deinococcus*– *Thermus*	predicting the prognosis of hepatocellular carcinoma after surgery	[89]

### Esophageal cancer

Esophageal cancer ranks sixth in terms of global cancer mortality and has two main subtypes, namely esophageal adenocarcinoma (EADC) and esophageal squamous cell carcinoma (ESCC) [90]. ESCC is the most prevalent histological subtype of EC worldwide [91, 92], and EADC is usually associated with Barrett’s esophagus [93]. Recently, several researchers have reported the changes in the microbiome inside tumors during the progression of EC and their influence on the cancer treatment.

Yamamura et al. (2016) quantified the presence of *F. nucleatum* DNA in 325 excised specimens of EC using quantitative polymerase chain reaction (qPCR). They observed a significant association between the presence of *F. nucleatum* and decreased survival rates [48]. Additionally, the prevalence of *F. nucleatum* was strongly correlated with the growth of ESCC tissues, indicating its potential significance in the progression of ESCC [52]. Furthermore, *F. nucleatum* modulated the expression of endogenous LC3 and ATG7 proteins and facilitated the formation of autophagosomes, which resulted in the development of chemoresistance against 5-fluorouracil, cisplatin, and docetaxel [51]. Notably, high *F. nucleatum* load was also associated with negative side effects during neoadjuvant chemotherapy [50]. The bacterial abundance varies during EC progression, and these alterations are associated with the clinical characteristics and prognosis of EC [28, 49]. Snider et al. observed variations in the microbial community associated with Barrett’s esophagus in patients with high-grade dysplasia and EADC [12]. These shifts were characterized by an increase in specific bacterial groups potentially associated with pathogenicity, such as Proteobacteria. Overall, these findings indicate that alterations in tumor microbiota are significantly associated with clinical prognoses and chemotherapy-related side effects in patients.

### Gastric cancer

Gastric cancer ranks fifth among the most prevalent types of cancer and is the fourth leading cause of cancer-related deaths worldwide [2, 94]. Chronic infection with *H. pylori* is considered the primary underlying factor of noncardia GC, and approximately all instances of this type of cancer are linked to this bacterium [95, 96]. However, the development of carcinoma of the cardia is not correlated with *H. pylori* infection and shows an inverse correlation in certain populations [97, 98].

Wang et al. (2015) evaluated a Chinese cohort and indicated that the primary impact of *H. pylori* on the microbial community is the increased bacterial load in the stomach rather than a change in the proportional abundances of non- *H. pylori* bacterial groups [53]. However, two years later, Liu et al. determined that the proportion of *H. pylori* in GC tissues obtained from patient groups in China and Mexico was lower than that in the adjacent nontumor tissues obtained from these patients. Moreover, the relative abundance of non-*H. pylori* bacteria was also altered in the tumor tissues, suggesting a potential alteration of the microbial composition in GC samples [39]. Subsequently, Ferreira et al. analyzed the gastric microbial community using 16 S rRNA sequencing and real-time qPCR [54]. The authors revealed the dysbiotic microbiota associated with GC, indicated by reduced microbial diversity, decreased abundance of *H. pylori*, and enrichment of other intestinal commensal bacteria. Furthermore, Liu et al. analyzed gastric microbiota by targeting the 16 S rRNA gene in a cohort of 276 patients with GC who had not received preoperative chemotherapy [55]. The authors proposed that the composition and diversity of the gastric microbiota were determined by specific stomach microhabitats associated with GC rather than being influenced by the stages or types of GC. Mannion et al. (2023) used the shotgun metagenomic approach to determine the microbial functional and composition variations of the microbiome within high-risk and low-risk cohorts of GC [56]. They claimed that it was possible to identify specific intratumoral microorganisms associated with GC by taking advantage of next-generation sequencing methods. Overall, these findings offer novel insights for the prophylaxis, therapy, and diagnosis of GC based on the associated microbiome.

### Colorectal cancer

Colorectal cancer is the third leading cause of cancer-associated morbidity, and despite a gradual reduction in mortality since 1978, it remains the second most prevalent cause of cancer-related fatalities in both males and females [2, 3]. Several authors have suggested a close association between CRC and the gut microbiota [99–101].

Several researchers have used novel identification methodologies such as qPCR, 16 S rRNA sequencing, and metagenomic analysis and reported the prevalence of *Fusobacterium* in individuals diagnosed with CRC [57–59]. Additionally, the concentration of *F. nucleatum* DNA in CRC tissue is negatively correlated with patient survival, ultimately resulting in shorter survival times [60, 64, 72]. In the research, this negative correlation was attributed to the ability of bacteria to promote nerve invasion, vascular tumor thrombus formation, and location of tumor [72]. Hamada et al. indicated that *F. nucleatum* modulated immune response within the tumor microenvironment [70]. The bacterium inhibited adaptive antitumor immune responses in MSI-High CRC, whereas showed pro-inflammatory effects in MSI-Low CRC. Moreover, increased *F. nucleatum* DNA within cancerous tissues was correlated with increased macrophage permeation and CDKN2A hypermethylation in MSI-High CRCs [66]. *F. nucleatum* impaired T-cell-mediated immune responses against colorectal tumors, thereby promoting the tumor growth. Therefore, *F. nucleatum* DNA can be considered a prognostic biomarker for CRC [64]. Thus, the administration of antibiotics targeting *F. nucleatum* may potentially affect the development and advancement of CRC [67, 74]. Interestingly, the load and abundance of microorganisms in the tumor may also vary depending on various factors, including the stage of cancer and location of tumor [61, 65, 73]. In addition to *F. nucleatum*, other bacteria may also influence the development, progression, and clinical manifestations of CRC [62, 63, 68, 77]. Coker et al. performed metabolomic and metagenomic analysis on stool samples from 386 subjects and demonstrated that bacterial metabolites, such as l-alanine, glycine, and l-proline, can promote the development of CRC and serve as markers for the non-invasive diagnosis of colorectal neoplasia [76].

The intratumoral enteric viruses, a critical constituent and regulator of the gut microbiota, influence the composition and abundance of intestinal microorganisms. Therefore, these viruses can influence the incidence, development, and outcomes of CRC [69, 99]. Furthermore, modifications in the composition of the intestinal fungal community were associated with CRC. The concentrations of *Malassezia* spp. increased, whereas those of *Saccharomyces* and *Pneumocystis* markedly declined in CRC tissue samples [71, 75].

### Pancreatic cancer

Pancreatic cancer is a malignant neoplasm with high mortality rates, where the incidence and mortality rates are almost similar [2]. The disease has a highly unfavorable prognosis and is the third most common cause of cancer-associated deaths in the United States [102, 103]. The presence of intratumoral microorganisms not only facilitates the onset and progression of PC but also influences treatment responsiveness and prognostication [11, 22].

Several authors have reported the colonization of the pancreas by multiple microorganisms both in healthy and diseased states [24, 80, 81]. However, the mechanisms by which microorganisms can infiltrate the supposedly aseptic pancreas are debatable. Nalluri et al. identified a considerable rise in the prevalence of Enterobacteriaceae in PC tissues among patients who underwent biliary stent implantation, suggesting that the biliary tract could potentially serve as a channel for microbial entry into the pancreas [25]. Kohi et al. found that the microbes in the duodenal fluid of adenocarcinoma patients were different from those in the duodenal fluid of healthy controls [87]. Moreover, microbial composition in PC tissues was similar to that in the duodenum, suggesting that bacteria may migrate from the intestine to the pancreas. Geller et al. also supported this hypothesis [80]. However, further studies are required to explore the mechanisms by which microorganisms gain access to the pancreas and determine whether these entry routes influence the progression and outcome of PC.

Several studies have reported that microbiota composition will significantly varies in response to a variety of influences [11, 82, 85–87]. Furthermore, several authors have determined that oral microbial communities may be implicated in the origin and progression of PC [79, 84]. The association of *H. pylori* with pancreatic ductal adenocarcinoma (PDAC) is controversial, although *H. pylori* is considered a risk factor for GC and can be found in cancer tissues. Nilsson et al. reported a detection rate of *H. pylori* DNA in 75% (30/40) of PC samples [23]. Conversely, Jesnowski et al. [78]. reported no evidence of *H. pylori* DNA in chronic pancreatitis or other PDAC tissue samples. In addition to bacteria, fungi have also been identified in PC tissue [104]. The findings reported by Aykut and colleagues provided confirmatory evidence for the enrichment of Malassezia in the fungal communities associated with PDAC [83]. Moreover, the study reveals that pathogenic fungi were enabled to activate mannose-bound lectins, which can promote tumorigenesis in the pancreatic environment.

### Hepatocellular carcinoma

Liver cancer ranks as the sixth most prevalent and the fourth most lethal neoplasm globally; its incidence is rapidly increasing worldwide [105–107]. HCC constitutes approximately 90% of the cases of liver cancer. Hepatitis B virus infection is a major risk factor for HCC, contributing to approximately 50% of reported cases [108].

The intestinal/hepatic axis establishes communication between the intestinal microbiota and the liver through the portal vein system. Microorganisms migrate from the intestine to the liver through the portal vein and may influence the pathological condition of the liver [109, 110]. Although the effect of gut microbiota on liver diseases has been extensively studied, the intratumoral microbiota of HCC remains unexplored [111–113]. In 2004, Huang et al. identified the 16 S rDNA of *H. pylori* in 8 out of 20 primary liver cancer samples; however, it was unclear whether these *H. pylori* strains were present within the tumor cells at that time [30]. In 2020, Sookonian et al. verified the presence of intrahepatic bacteria DNA in hepatocytes. They conducted high-throughput sequencing analysis of the 16 S rRNA gene in liver tissues obtained from 97 obese patients with NAFLD and 19 non-obese patients with NAFLD (control group) [114]. Their results indicated that the bacterial DNA profiles of the liver of morbidly obese patients with NAFLD differed significantly from those of non-obese patients with NAFLD. Nevertheless, experimental evidence for the presence of microorganisms in liver cancer cells is yet to be established. Interestingly, Huang et al. observed a notable increase in the levels of microbial α- and β-diversities of both the peritumor area and HCC tissue compared to the normal controls [88]. He et al. have discovered that the microbial diversity within HCC tissue is significantly higher than its surrounding tissues, as determined by a thorough analysis [115]. Moreover, they investigated the underlying mechanism by which microbial communities affect lipid metabolism and their impact on HCC progression. Recently, Sun et al. reported significant heterogeneity in the intratumoral microbiome of HCC [89]. They used microbial profile-based clustering and developed a hepatotype, which may serve as an independent biomarker for the prediction of HCC prognosis after surgery.

## Effect of intratumoral bacteria on digestive system tumors

**Fig. 3:**
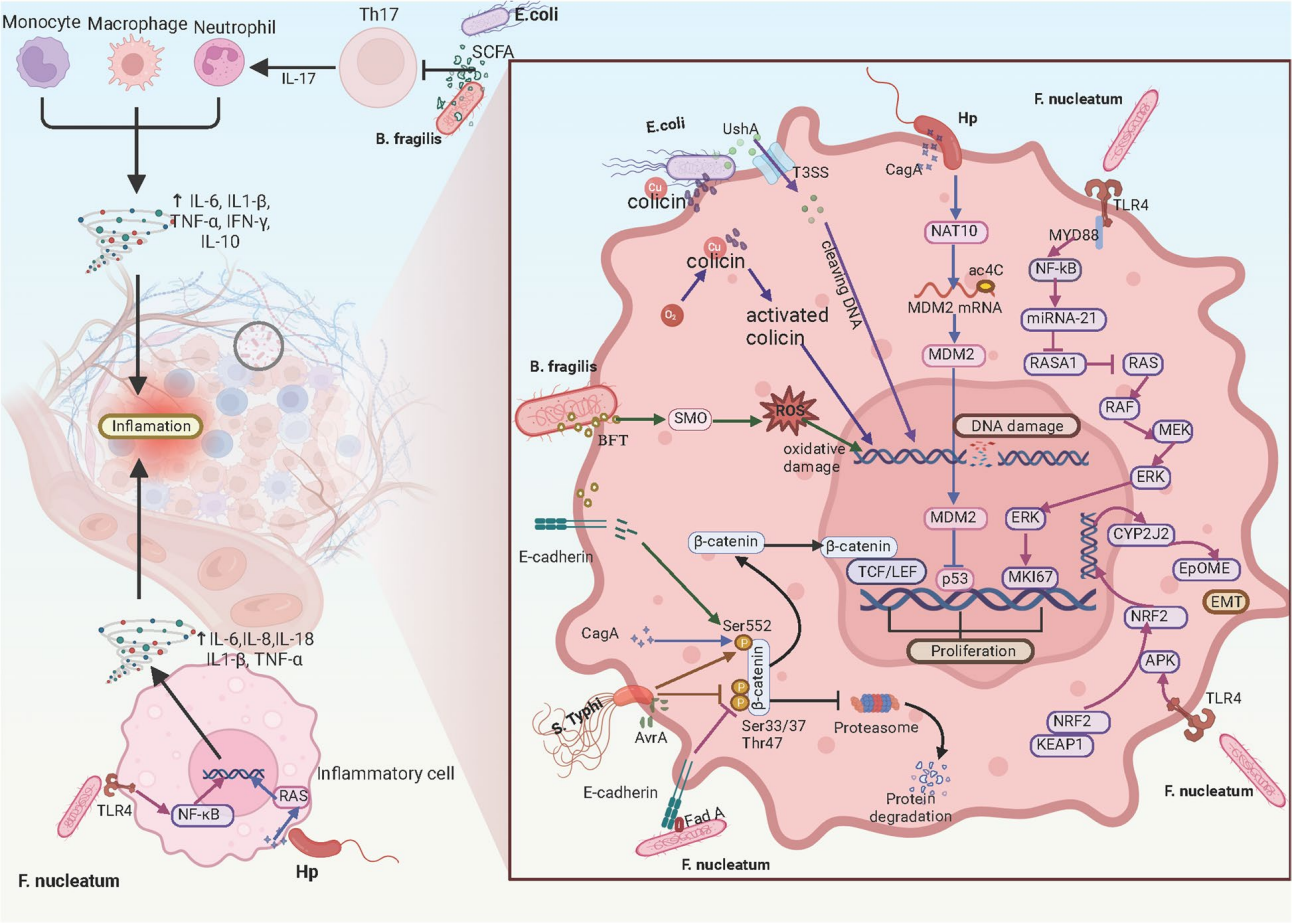
Effect of intratumoral bacteria on digestive system tumors. Bacteria can modulate diverse signaling pathways or initiate DNA damage leading to carcinogenesis in cells. Chronic inflammation constitutes a notable risk factor for tumor development. Bacteria may influence inflammatory cells and can directly induce inflammation, or they may regulate immune cells and inhibit inflammation

Microorganisms are involved in the initiation, progression, and inhibition of tumors in the digestive tract [116–118]. Owing to their distinct physiological characteristics, different microbes manifest differential effects on tumor survival, proliferation, and invasion [119]. The microbial communities of digestive system tumors show considerable diversity. Herein, we elaborate on the effects of specific bacteria on digestive system tumors and the respective mechanisms underlying these effects (Table 2; Fig. 3).

**Table 2:** Regulatory effect of typical intratumoral bacteria on digestive system tumors

Bacterium	Regulator	Signaling pathway	Target	Regulation	Expression/Activity	Effect	Neoplasm promotion/suppression	Refs
*Bacteroides fragilis*	BFT	/	Interleukin-17	Positive	↑	DNA damage	promotion	[68]
	BFT	/	Spermine oxidase	Positive	↑	DNA damage	promotion	[120]
	BFT	β-catenin	E-cadherin	Negative	↓	tumor cell proliferation	promotion	[121]
	BFT	β-catenin	E-cadherin	Negative	↓	inducing c-myc expression	promotion	[122]
	BFT	NF-κB	ERK1/2, p38, and JNK kinases	Positive	↑	inducing intestinal inflammation and mucosal damage	promotion	[123]
	BFT	NF-κB	IκB	Negative	↓	inducing neutrophil migration	promotion	[124]
	BFT	NF-κB	Interleukin − 8	Positive	↑	inducing intestinal inflammation	promotion	[125]
	BFT	MAPK	human beta-defensin-2	Positive	↑	suppressing intestinal inflammation	suppression	[126]
	butyrate	nod-like receptor signaling pathway	Interleukin − 18 and Interleukin − 1β	Negative	↓	suppressing intestinal inflammation	suppression	[127]
	PSA	TLR-2	Interleukin − 10	Positive	↑	suppressing intestinal inflammation	suppression	[128]
	PSA	Interleukin − 12/Stat4	Th1	Positive	↑	inducing Th1 cytokine production	suppression	[129]
*Escherichia coli*	colibactin	/	Interleukin-17	Positive	↑	DNA damage	promotion	[68]
	colibactin	/	adenine	Negative	↓	DNA damage	promotion	[130–133]
	colibactin	/	copper	Positive	↑	DNA damage	promotion	[134]
	T3SS	/	UshA	Positive	↑	DNA damage	promotion	[135]
	VirF	/	PV-1	Positive	↑	gut vascular barrier damage	promotion	[34]
	acetic acid	/	Interleukin-1β, Interleukin-6, Interleukin-8, TNF-α	negative	↓	suppressing intestinal inflammation	suppression	[136]
	/	PI3K-AKT	PTEN	Negative	↓	inducing apoptosis	suppression	[137]
*Fusobacterium nucleatum*	/	TLR4	microRNA-21	Positive	↑	increasing tumor proliferation	promotion	[138]
	/	TLR4	12,13-EpOME	Positive	↑	promoting EMT and metastasis	promotion	[139]
	FadA	β-catenin	E-cadherin	Positive	↑	inducing intestinal inflammation	promotion	[140]
	/	NF-κB	PTGS2, Interleukin-1β, Interleukin-6, Interleukin-8, TNF-α	Positive	↑	inducing intestinal inflammation	promotion	[37]
	/	/	CCL20	Positive	↑	increasing tumor invasion	promotion	[48]
	/	/	CD3 + T	Negative	↓	suppressing antitumor immune response	promotion	[141]
	Fap2	/	TIGIT	Positive	↑	inhibiting NK cell cytotoxicity	promotion	[142]
	Fap2	/	CEACAM1	Positive	↑	inhibiting NK cell cytotoxicity	promotion	[143]
*Helicobacter pylori*	CagA	β-catenin	/	/	/	increasing β-catenin nuclear accumulation	promotion	[144]
	CagA	β-catenin	PPARδ	Positive	↑	inducing tumor proliferation	promotion	[145]
	CagA	NF-κB	Interleukin-8	Positive	↑	inducing intestinal inflammation	promotion	[146]
	/	/	N-acetyltransferase-10	Positive	↑	increasing mRNA acetylation	promotion	[147]
*Salmonella typhi*	AvrA	β-catenin	phosphorylated-β-catenin	Positive	↑	increasing nuclear accumulation of β-catenin	promotion	[148–150]

### Bacteroides fragilis

*B. fragilis* is a gram-negative anaerobic bacterium, primarily inhabiting the intestinal tract [151]. *B. fragilis* is an opportunistic pathogen comprising two distinct groups, namely non-toxigenic *Bacteroides fragilis* (NTBF) and enterotoxigenic *Bacteroides fragilis* (ETBF) [152]. NTBF can cause limited intestinal damage [153], whereas ETBF causes intestinal mucosal injury and inflammation by secreting BFT, a 20 kDa zinc-dependent metalloproteinase toxin [33, 153, 154]. Notably, intestinal tissue inflammation and damage can induce carcinogenesis, particularly CRC [152, 155].

The exposure of the mucosal tissue to BFT has been strongly implicated in the pathogenesis of CRC. Boleij et al. indicated that BFT exposure may constitute a significant risk factor for the development of CRC [33]. The authors also suggested that the incidence of BFT detection is significantly higher in patients with advanced-stage CRC compared with those with early-stage disease. Subsequently, several authors have investigated the precise mechanisms by which BFT promotes the development of CRC. The co-colonization of *B. fragilis* and *E. coli* induced interleukin (IL)-17 secretion, leading to colonic epithelial DNA damage [68]. Consequently, tumor onset was accelerated and mortality rates increased in patients with CRC. Goodwin et al. reported that BFT upregulated the expression of spermine oxidase (SMO), and reactive oxygen species (ROS), formed as a byproduct of spermine oxidation, lead to DNA damage [120]. They also observed that SMO mediated by BFT can increase the generation of SMO-dependent ROS and γ-H2A.x (a DNA damage marker). The accumulation of DNA damage promoted the uncontrolled growth of epithelial cells, eventually progressing into CRC [156]. However, Snezhkina et al. performed qPCR analysis and revealed that SMO and BFT were not significantly correlated with each other. In addition, BFT can induce proteolytic degradation of E-cadherin in the E-cadherin/β-catenin complex, consequently facilitating the nuclear translocation of β-catenin and resulting in the malignant proliferation of cells [121, 122]. The nuclear factor (NF-κB) pathway is a prototypical pro-inflammatory signaling mechanism [157]. Bacterial effector protein BFT can exert a negative effect on IκB and consequently trigger the activation of the NF-κB pathway, resulting in the induction of tumorigenesis [123–125].

In contrast, some investigators have reported that *B. fragilis* can exert antitumor effects. The bacterium can induce the upregulation of human β-defensin-2 gene expression by activating the MAPK pathway. Notably, the insufficient expression of this gene in the intestine increase susceptibility to ETBF-associated diseases, including colitis, irritable bowel disease, and colon tumorigenesis [126]. Furthermore, *B. fragilis* exerts a negative effect on the NLRP3-mediated pathways of inflammatory signaling by stimulating the secretion of butyrate [127]. This compound inhibits macrophage activation and secretion of pro-inflammatory mediators such as IL-18 and IL-1β, thereby reducing the levels of intestinal inflammation and restricting the development of CRC. *B. fragilis* can also impair tumor formation and invasion by activating CD4 + T cells, thereby inducing the production of anti-inflammatory molecules such as IL-10 [128, 129]. Taken together, these pathways can be targeted for the development of novel therapeutic regimens.

### Escherichia coli

*E. coli* is a facultatively anaerobic gram-negative bacterium commonly present in the normal intestinal microbiota [158]. Most *E. coli* strains are commensals and rarely cause diseases in their host. Nevertheless, specific strains can produce toxins having genotoxic properties, which can regulate cell differentiation, apoptosis, and proliferation [159–161]. The possible role of these toxins in either stimulating tumor development or inhibiting tumor progression is an active area of cancer research.

*E. coli* possesses the pathogenic polyketide synthase (pks) genomic island, which encodes a cluster of enzymes responsible for synthesizing colibactin [130]. This peptide alkylates DNA at the adenine residues [131, 132], leading to double-stranded breaks in the host DNA and cell-cycle arrest [130]. Consequently, the uncontrolled proliferation of cells leads to malignancy [133]. Arthur et al. indicated that the removal of the pks genotoxic island from *E. coli* NC101 reduced tumor occurrence and invasiveness in mice, whereas no changes were observed in intestinal inflammation [162]. Liu et al. suggested that the pathogen engages with the intestinal epithelial cells through its type III secretion system and translocates the UshA protein [163]. UshA is a potent genotoxin that can degrade the intestinal epithelial cell DNA, ultimately leading to carcinogenesis [135]. Furthermore, *E. coli* can induce double-stranded breaks in the host DNA through copper-mediated oxidative cleavage and has been shown to promote DNA damage by inducing IL-17, ultimately resulting in tumorigenesis [68, 134]. Colibactin interacts with exchangeable copper in the gut to create a complex that coordinates with oxygen in epithelial cells to produce activated colibactin, which attacks and cleaves DNA [134]. In addition, the onset of CRC facilitates the colonization of *E. coli* in the hepatic tissues by impairing the integrity of the intestinal vascular barricade through virulence regulator VirF, consequently leading to liver metastases of CRC [34].

In contrast, *E. coli* also has anti-inflammatory and anticancer properties. Nakkarach et al. reported that *E. coli* secretes short-chain fatty acids (SCFA), which can inhibit the production of pro-inflammatory mediators, such as IL-1β, IL-6, IL-8, and TNF-α [136]. This downregulation may result in the inhibition of cancers linked to inflammation. However, the authors mainly examined the effect of bacteria on breast cancer, and further studies are required to evaluate their effect on CRC cells. *E. coli* elicits pro-apoptotic effects on CRC cells by upregulating PTEN and AKT1 [137]. Dalmasto et al. proposed an alternative perspective on the contribution of pks + *E. coli* in the pathogenesis of CRC [164]. The researchers infected xenografts with pks + *E. coli* at a multiplicity of infection of 100 and did not detect any obvious pro-proliferative effect. Moreover, they observed a reduction in tumor growth. Therefore, further investigations on the role of *E. coli* in tumor development hold significant potential in CRC diagnostics and therapeutics.

### Fusobacterium nucleatum

F. nucleatum is an anaerobic, gram-negative, and opportunistic bacterium that colonizes both the gastrointestinal and oral tracts [165]. F. nucleatum can attach to and infiltrate endothelial and epithelial cells through its virulence factors, including adhesin A (FadA), Fusobacterium autotransporter protein 2 (Fap2), and fusobacterial outer membrane protein A (FomA) [26, 140, 142, 166]. Several authors have indicated a possible connection between *F. nucleatum* and the onset of carcinogenesis.

*F. nucleatum* can promote the initiation, proliferation, and progression of tumors by eliciting an inflammatory response and suppressing the anticancer immune response. *F. nucleatum* regulates microRNA-21 and cytochrome P450 monooxygenases, mainly CYP2J2, as well as its mediated product 12,13-EpOME through the TLR4 signaling pathway to facilitate the epithelial–mesenchymal transition and tumor invasion [138, 139]. Notably, patients with high microRNA-21 expression tend to have shorter survival times compared to those with low micro-RNA-21 expressions [139]. Additionally, *F. nucleatum* exerts its modulatory effect on the tumor immune microenvironment and promotes tumor progression by inducing NF-κB-mediated inflammation, which, in turn, facilitates the recruitment of myeloid-derived suppressor cells to the tumor microenvironment [37]. This recruitment is accompanied by inhibition of T-cell proliferation and induction of T-cell apoptosis, further underscoring the role of *F. nucleatum* in promoting tumor growth [141].

Immune checkpoints play a critical role in regulating the immune response and facilitating T-cell dysfunction in autoimmunity and inflammation [167, 168]. Nonetheless, these inhibitory pathways can be co-opted by neoplastic cells to facilitate tumor immune evasion [169, 170]. Gur et al. reported that the Fap2 outer surface protein of *F. nucleatum* curtailed antitumor immunity by binding to and inducing activation of inhibitory receptors, i.e., T-cell immunoreceptor with immunoglobulin and ITIM domain (TIGIT) and carcinoembryonic antigen-related cell adhesion molecules 1 (CEACAM1), which are expressed by T and natural killer cells [142, 143]. Interestingly, *F. nucleatum* triggers distinct immunological reactions in CRC cases with different MSI statuses [70]. The activation of STING signaling by *F. nucleatum* resulted in increased PD-L1 expression and accumulation of interferon-gamma and subsequently CD8 + tumor-infiltrating lymphocytes, leading to tumor inhibition [171]. Such effects, when combined with PD-L1 blockade treatment, enhanced tumor sensitivity and response to the immune checkpoint blockade, leading to a marked improvement in the overall survival rates of patients [171]. Overall, investigating the precise mechanism underlying the role of *F. nucleatum* in the development, invasion, and suppression of tumors will lead to the development of new therapeutic interventions for gastrointestinal cancers.

### Other microorganisms

Apart from the bacteria mentioned above, other microorganisms also exert a distinct influence on the metabolism or microenvironment of tumors. *H. pylori* is a gram-negative bacterium that can selectively colonize gastric epithelium and is associated with the development of digestive system tumors [172]. The presence of *H. pylori* in gastrointestinal tumors may induce inflammation, thereby facilitating the proliferation of malignant cells through the activation of β-catenin or NF-kB pathways [144–146]. Moreover, *H. pylori* may contribute to the progression of GC through the upregulation of NAT10 expression, which subsequently stabilizes MDM2 mRNA [147]. Furthermore, *Salmonella typhi* can enhance the proliferation of tumors in the digestive system by activating the β-catenin pathway [148–150].

## Potential applications of intratumoral bacteria in digestive system tumors

### Diagnostic value of intratumoral bacteria

Intratumoral microorganisms have great potential as independent diagnostic markers because of their richness and heterogeneity in different tumors [79, 173–175], as well as their varying composition at different stages of the same tumor [12, 39]. For example, altered microbiome composition in Barrett’s esophagus, characterized by a decrease in the abundance of Firmicutes and an increase in the abundance of Proteobacteria, has been associated with high-grade dysplasia and esophageal adenocarcinoma [12]. Therefore, microbiota alterations in Barrett’s esophagus may be tracked for diagnosing esophageal cancer. In addition, an increase in the abundance of *Porphyromonas gingivalis* and *Aggregatibacter actinomycetemcomitans* has been linked to an increased probability of developing PC [79]. Therefore, multiple diagnostic approaches are recommended to detect any increase in the concentrations of these specific bacteria in pancreatic tissue, thereby minimizing the risk of missing the diagnosis of PC. Furthermore, in addition to the oral microbiota, the presence of fungal genera, such as *Malassezia*, can also be considered for detecting PC [83, 176]. Overall, identifying the microbial composition and alterations in pathological tissues can be used as an adjunct approach to the diagnosis of cancer.

### Therapeutic effects of intratumoral bacteria

**Fig. 4:**
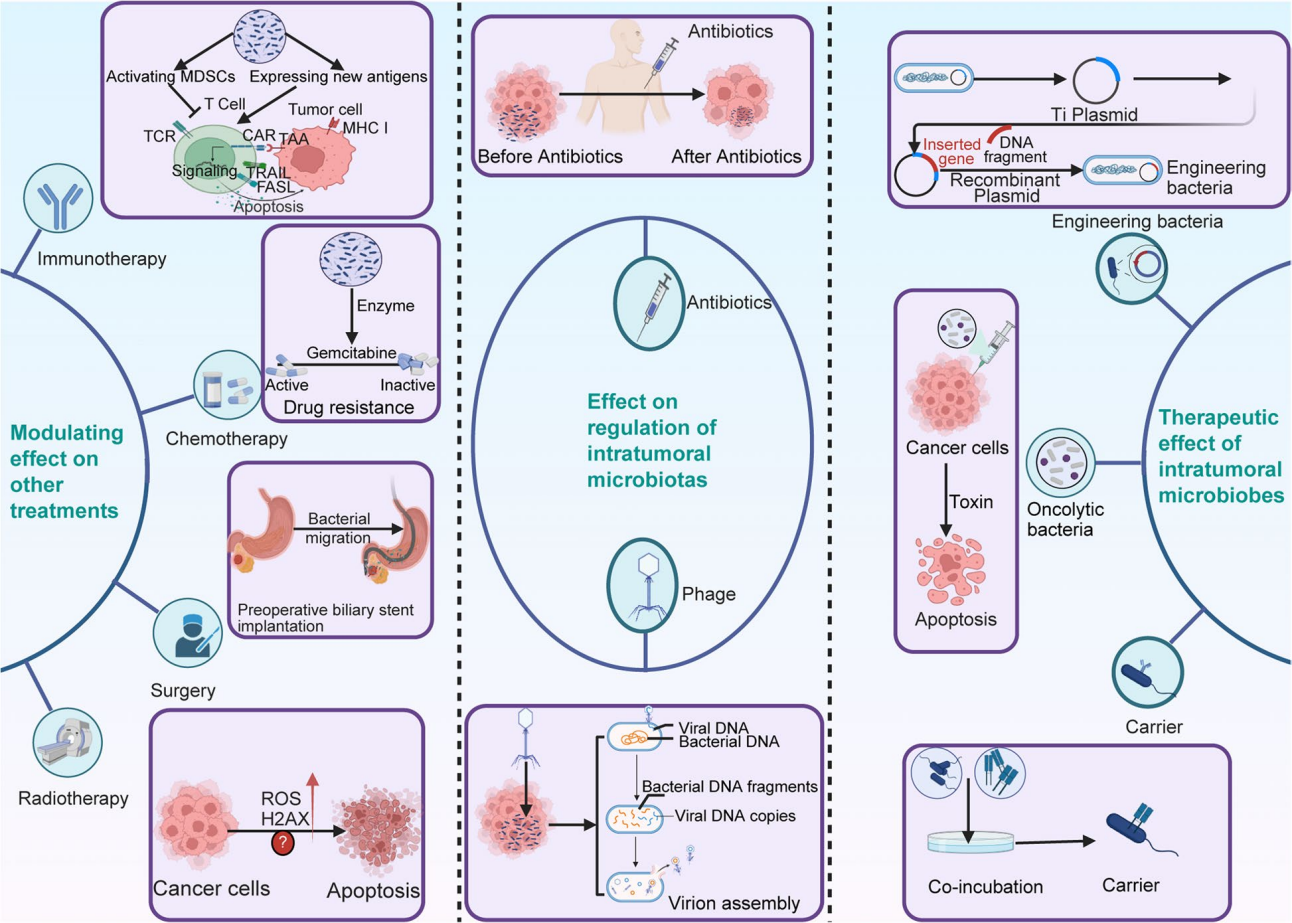
Therapeutic effect of intratumoral bacteria. Bacteria can promote or inhibit the effects of immunotherapy on the host by regulating immune cells. Bacteria possess oncolytic properties. They may serve as vectors or can be genetically modified to facilitate targeted therapeutics. Antibiotics and bacteriophages can modify the composition of intratumoral bacteria, offering potential therapeutic options for the prevention and treatment of cancer

Despite rapid advancements in cancer diagnosis and treatment, the global burden of cancer-related deaths is rapidly increasing [2]. According to data published by WHO in 2019, cancer remained a leading cause of death before the age of 70 in most countries worldwide. Microorganisms have functional significance in the initiation and progression of gastrointestinal tumors; therefore, it is imperative to examine their potential use as therapeutic tools in cancer management (Fig. 4).

#### Intratumoral microorganisms influence anticancer treatment

Currently, the modalities for cancer management include surgical intervention to excise neoplastic tissues, radiotherapy to inhibit the proliferation of tumor cells, and chemotherapy to kill malignant cells by inducing cytotoxic mechanisms or using direct chemical agents [177]. Immunotherapy has emerged as a novel approach, which boosts the immune response of the host for recognizing and eliminating tumor cells, resulting in promising therapeutic outcomes [178]. Intratumoral microorganisms can modulate the immune response within the tumor microenvironment, and consequently, these microbes can influence the effectiveness of immunotherapies for digestive system tumors [173].

Nalluri et al. evaluated intratumoral microorganisms in PC and revealed that individuals who underwent Whipple surgery or preoperative biliary stent implantation showed increased bacterial colonization in the pancreas [25]. However, the authors were not able to determine the correlation between these bacteria and the prognosis of PC. Furthermore, the influence of intestinal bacteria and fungi on the response to radiotherapy has been observed in murine models of breast cancer and melanoma [179]. Nevertheless, it remains uncertain whether these gut microorganisms are present in the digestive system tumor tissues or they exert a significant effect on the response of tumors to radiotherapy. Chemotherapy resistance occurs in neoplastic tissues partly due to the metabolic activities of intratumoral microorganisms [180]. Geller et al. suggested that intratumoral bacteria in PC can produce enzymes that metabolize the chemotherapeutic drug gemcitabine, rendering cancer cells resistant to the drug [181]. The aforementioned discovery has been thoroughly substantiated through a multitude of experimental trials [182, 183].

Introtumoral microorganisms play a positive or negative role in anti-tumor immune responses by mediating diverse immune cells and PD-1/PD-L1 axis [184, 185]. Numerous literatures have reported the facilitating role of intratumoral microorganisms in the anti-tumor immune responses. For example, *Lachnospiraceae* family bacteria residing in normal tissues of patients with CRC can degrade lysoglycerophospholipids which injury CD8^+^ T cell activity, thereby controlling the development of CRC by improving immune tumor immune surveillance [186]. *E. coli* is a common bacterium isolated from solid tumors including CRC. *E. coli TOP10* induces the activation of CD8^+^ and CD4^+^, which are the effector cells to inhibit tumor occurrence and progression [187]. Shi et al. observed that systemic administration and local delivery of *Bifidobacterium* promote innate immune responses in mice with CRC [188]. Additionally, depletion of microorganisms in PDAC upregulates the expression of PD-1, which leads to positive effects on immunotherapy targeting PD-1 [22]. However, intratumoral microorganisms can impede the efficacy of anti-tumor immunotherapy. Commensal bacteria enhance the generation of immunosuppressive Treg cells and stimulate the growth of cancerous cells via metabolites such as butyrate [189, 190]. Zhang et al. discovered a correlation between intratumoral bacteria and the proportion of PD-L1 epithelial cells. Moreover, their findings illustrated that elevated levels of intratumoral *Lactobacilli* contributed to the formation of an immunosuppressive tumor microenvironment, which predicted the poor prognosis of ESCC [191].

#### Intratumoral microorganisms show therapeutic effects on cancer

Goto et al. identified the presence of intratumoral lytic bacteria that can exert anticancer effects [192]. Bacteria have high targeting properties and minimal toxicity, making them viable delivery vectors [177]. *Listeria* species, co-incubated with cytotoxic and labeled antibodies, can specifically target and lyse PC cells, thereby inhibiting cancer metastasis [193]. Interestingly, the use of *Salmonella* strains expressing Fas ligands has demonstrated notable efficacy in inducing antineoplastic responses against colon cancer [194].

Genetic engineering techniques have enabled us to modify bacterial genomes to enhance their safety, antitumor activity, and carrier function to fulfill the unique requirements of complex pathological environments. These genetically engineered bacteria have been used for treating several tumors. The attenuated strain of *Salmonella typhimurium* VNP20009 has shown significant anticancer properties in several animal models of cancer [195]. The bacteria negatively affected the growth of PC by inducing severe necrosis and apoptosis in a dose-dependent manner [196]. The engineered microorganisms, serving as vectors, can penetrate necrotic and hypoxic regions within tumor tissue, which is beyond the reach of normal bacteria [197]. In addition, engineered bacteria expressing immunomodulatory factors can to potentiate the immune response mediated by immune cells and cytokines, thereby inhibiting the progression and invasion of malignant tumors. Zheng et al. reported that engineered bacteria secreting FlaB can stimulate the infiltration and differentiation of immune cells via Toll-like receptors, suppressing the growth and metastasis of colon cancer [198].

#### Targeting intratumoral microorganisms for cancer treatment

The majority of intratumoral microorganisms inhibit the antitumor response and instead promote tumor proliferation [22, 47]. Therefore, eliminating intratumoral microorganisms may be a potential adjunct anticancer treatment. Bullman et al. administered metronidazole to mice hosting colon cancer xenografts and found a decrease in *Fusobacterium* load and subsequent inhibition of cancer cell proliferation and overall tumor growth [67]. The administration of a nonsteroidal anti-inflammatory drug, namely aspirin, has been reported to show direct antibacterial activity against *F. nucleatum* and decrease the prevalence of *F. nucleatum* in CRC [74]. Moreover, the treatment distinctly inhibited the promotion of intestinal tumorigenesis by *F. nucleatum*. Apart from bacteria, the protective effect of fungi on the progression of PDAC has been demonstrated in mouse models, indicating that targeting the mycobiome could be a promising avenue for cancer treatment [38].

However, antibiotics are not selective for intratumoral bacteria, thereby limiting their potential antitumor effects. In contrast, bacteriophages are highly specific for their target bacteria and can precisely lyse intracellular microbes [199]. Scientists injected bacteriophages specifically targeting *F. nucleatum* into the mice and found that bacteriophages were able to penetrate tumor tissue and infect their target bacterium [200]. Zheng et al. used phage-modified nanoparticles for intravenous or oral administration in mice suffering from CRC [201]. The authors found an enhanced chemotherapy effect and a reduction in the *F. nucleatum* load. Taken together, bacteriophages precisely target specific intratumoral bacteria and eliminate them, thereby providing a meticulous approach to cancer treatment.

Apart from eliminating intratumoral microorganisms through the aforementioned pathways, manipulating the composition of the microbiota accomplished by fecal microbiota transplantation is also an effective strategy to inhibit cancer growth [47]. Fecal microbiota transplantation bolsters the body’s immune response against tumors and effectively inhibits the progression of patients with CRC [202]. Consequently, therapeutic interventions focused on intratumoral microorganisms exhibit significant potential in enhancing the overall prognosis of individuals diagnosed with digestive system tumors.

### Prognostic potential of intratumoral bacteria

A statistically significant association exists between intratumoral microorganisms and survival and mortality rates in patients with cancer [191, 203]. Therefore, the presence of intratumoral microorganisms can be used as a prognostic tool for predicting the outcome in cancer patients. The mucosal microbiome exhibits dynamic association with CRC and thus can be explored for developing microbiota-based prognostic approaches for CRC [61]. The prevalence of *F. nucleatum* simultaneously increases with tumor progression [60]. Additionally, a higher concentration of *F. nucleatum* DNA in CRC tissue is linked to decreased survival rates [64]. *F. nucleatum* has been historically linked to unfavorable prognostic outcomes and is significantly associated with negative prognosis in colorectal carcinomas, as well as esophageal and pancreatic malignancies [32, 48, 50]. However, Oh et al. challenged this assumption by showing that the influence of *F. nucleatum* on the prognosis of CRC depends on other determining factors [204]. They indicated that the positive prognostic impact of *F. nucleatum* was solely detected in sub-categories of non-sigmoid carcinoma patients with high levels of non-MSI. Therefore, they concluded that the position of the tumor and the combined status of MSI may play a crucial role in influencing the diverse prognostic impact of *F. nucleatum* in patients with CRC undergoing adjuvant chemotherapy. In addition, several other microorganisms are closely associated with the prognosis of gastrointestinal malignancies. *H. pylori* releases CagA through its type IV secretion system, thereby resulting in increased susceptibility and unfavorable clinical outcomes in patients diagnosed with gastric or colorectal cancers [205, 206]. *Streptococcus* and *Prevotella* are more abundant in patients with ESCC having lymph node metastasis [49]. The presence of these microorganisms may be an independent predictive indicator for the prognosis of patients with ESCC. Overall, the detection of microorganisms in digestive system tumors can predict the prognosis of patients, thereby guiding timely modifications in treatment strategies for achieving favorable outcomes in affected patients.

Intratumoral microorganisms as tumor biomarkers exhibit significant potential in advancing the development of more efficacious therapeutic approaches and prognosis prediction models. Hermida et al. reported that RNA-seq and whole-genome sequencing provided by The Cancer Genome Atlas documented not only extensive information on thousands of cancer cases but also genetic information from intratumoral microorganisms [207]. Furthermore, they suggested that combining information about tumor gene expression and differences between the microbes in the tumor and those in non-tumor tissue can predict cancer prognosis and drug response. Additionally, Sun et al. indicated hepototype distinguished by differences in intratumoral microorganisms is demonstrated to be an independent prognostic factors for patients with postoperative HCC [89]. In their study, high level of *Akkermansia* and *Methylobacterium* is associated with favorable prognosis and can be used to construct clinical predictive models.

## Conclusion

Currently, intratumoral microorganisms are being actively investigated in several types of tumors and some remarkable results have been obtained in this context. Their diversity and functions have been described in detail using next-generation sequencing technology. Here, we reviewed the fundamental features, sources, types, and heterogeneity of microorganisms present in digestive system tumors. In addition, we summarized the role of some typical intratumoral microorganisms present in specific tumors. Finally, we elaborated on the potential use of tumor-associated microorganisms for developing novel diagnosis and treatment strategies for digestive system tumors. This information will be valuable for further investigating the role of microorganisms in neoplastic growth and developing microbial therapy for neoplasms.

## Data Availability

No original datasets were generated for this review. All data supporting the information given here can be found in the references cited within the paper.
